# Polyphosphate Kinase 2 (PPK2) Enzymes: Structure, Function, and Roles in Bacterial Physiology and Virulence

**DOI:** 10.3390/ijms23020670

**Published:** 2022-01-08

**Authors:** Nolan Neville, Nathan Roberge, Zongchao Jia

**Affiliations:** Department of Biomedical and Molecular Sciences, Queen’s University, Kingston, ON K7L 3N6, Canada; n.neville@queensu.ca (N.N.); 15nar2@queensu.ca (N.R.)

**Keywords:** polyphosphate, kinase, PPK2, virulence, *Pseudomonas aeruginosa*, inhibitor

## Abstract

Inorganic polyphosphate (polyP) has been implicated in an astonishing array of biological functions, ranging from phosphorus storage to molecular chaperone activity to bacterial virulence. In bacteria, polyP is synthesized by polyphosphate kinase (PPK) enzymes, which are broadly subdivided into two families: PPK1 and PPK2. While both enzyme families are capable of catalyzing polyP synthesis, PPK1s preferentially synthesize polyP from nucleoside triphosphates, and PPK2s preferentially consume polyP to phosphorylate nucleoside mono- or diphosphates. Importantly, many pathogenic bacteria such as *Pseudomonas aeruginosa* and *Acinetobacter baumannii* encode at least one of each PPK1 and PPK2, suggesting these enzymes may be attractive targets for antibacterial drugs. Although the majority of bacterial polyP studies to date have focused on PPK1s, PPK2 enzymes have also begun to emerge as important regulators of bacterial physiology and downstream virulence. In this review, we specifically examine the contributions of PPK2s to bacterial polyP homeostasis. Beginning with a survey of the structures and functions of biochemically characterized PPK2s, we summarize the roles of PPK2s in the bacterial cell, with a particular emphasis on virulence phenotypes. Furthermore, we outline recent progress on developing drugs that inhibit PPK2 enzymes and discuss this strategy as a novel means of combatting bacterial infections.

## 1. Introduction: Inorganic Polyphosphate and Polyphosphate Kinase 1 (PPK1)

Inorganic polyphosphate (polyP) is an ancient and evolutionarily conserved biopolymer consisting of phosphate monomers linked together via high-energy phosphoanhydride bonds. PolyP chains can range in length from three to over one thousand inorganic phosphate (P_i_) residues [1]. Insoluble phosphate-containing granules—what we now know to be polyP—have been documented in bacteria since the late 1800s [2]. However, the source of this mysterious phosphate polymer remained unclear until Arthur Kornberg and colleagues isolated an enzyme from *Escherichia coli* that could synthesize polyP, which they termed polyphosphate kinase (PPK; later disambiguated as PPK1) [3]. It was not until 1990 that *E. coli* PPK1 was finally purified to homogeneity [4], which allowed for the identification and cloning of the *ppk1* gene, followed by overexpression and deletion studies [5,6,7]. The newfound ability to manipulate the *ppk1* gene was a boon for polyP research. *E. coli* mutants lacking *ppk1*—and thus generating no detectable polyP—exhibited profound defects in stationary phase survival upon nutrient downshift [7,8,9,10,11]. PolyP has also been shown to protect bacteria from a variety of external stressors, such as heat [12], ultraviolet irradiation [13], antibiotics [14,15], metal ions [16], and oxidative stress [17,18]. Although it is not yet completely understood, at least some of these protective effects may be a result of chaperone-like function, whereupon polyP binds partially unfolded or denatured proteins to facilitate refolding once stress has abated [19].

Despite the absence of either PPK1 or PPK2 homologues in higher eukaryotes [20], it is worth noting that polyP has been detected in various mammalian cell types at concentrations typically ranging from 25 to 120 µM [21], though this can reach approximately 1 mM in platelets [22]. While the source of this mammalian polyP is unclear, it has been postulated that the mitochondrial F_0_F_1_ ATPase is involved [23]. PolyP has been linked to diverse aspects of mammalian physiology, including blood clotting [24], bone formation [25], and protein polyphosphorylation [26,27,28]. PolyP also serves as a nucleation source for amyloid proteins ranging from human α-synuclein and Tau to the *E. coli* biofilm protein CsgA [1,29]. Finally, several recent studies have demonstrated that bacterial polyP can modulate the mammalian immune response [30,31], suggesting an intriguing potential for host–pathogen polyP crosstalk during infection [32].

## 2. PPK2: A New Class of PolyP-Metabolizing Enzyme

**Figure 1:**
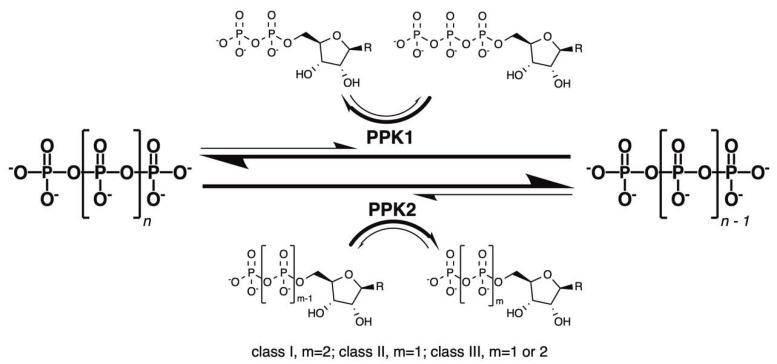
Comparison of the reactions catalyzed by PPK1 and PPK2 enzymes. R, nucleobase.

While most of the early work on bacterial polyP used *E. coli* (which encodes a single PPK1) as a model organism, other bacterial species such as *Pseudomonas aeruginosa* were eventually examined. The Kornberg lab observed that their ∆*ppk1* knockout strain of *P. aeruginosa* still produced up to 20% of wildtype polyP levels [33,34], in contrast to ∆*ppk1 E. coli* in which polyP is absent [6]. The source of this remaining polyP was later found to be a new class of enzyme termed PPK2 [34,35]. Remarkably, PPK2 shares no significant sequence identity with PPK1 [35]. Subsequent X-ray crystal structures of PPK2s from several species also corroborated a distinct structural fold relative to that of PPK1 [36]. On a functional level, *P. aeruginosa* PPK2 displayed a 75-fold preference for polyP degradation relative to synthesis, in contrast to PPK1 [34] (Figure 1). It therefore became clear that PPK1 and PPK2 were separate entities with distinct enzymology.

Despite the absence of homology between PPK1 and PPK2, both respective classes of enzymes are highly conserved throughout the bacterial kingdom [35,37]. Interestingly, it is postulated that PPK2 is evolutionarily older than PPK1 based on phylogenetic analysis and trends in amino acid loss and gain [38]. *ppk2* genes have been identified in many species of both Gram-positive and Gram-negative bacteria [35], with several species encoding multiple distinct isoforms [35,39,40] (Table 1). For example, *P. aeruginosa* possesses three distinct PPK2s [39], whereas other pathogenic bacteria such as *Mycobacterium tuberculosis* [41] or *Klebsiella pneumoniae* [42] possess only a single PPK2 isoform. In a more extreme instance, the environmental bacterium *Ralstonia eutropha* contains five distinct PPK2 enzymes, further illustrating the genetic abundance and variability among PPK2s [40] (Table 1). By contrast, bacteria rarely encode more than one *ppk1* allele [35,40,42].

The tendency of bacteria to encode one or more PPK2 isoform has in turn complicated the study of polyP physiology. *E. coli* does not encode any PPK2s, and yet has served as the principal model species for studying bacterial polyP dynamics. As a result, our understanding of polyP dynamics in species other than *E. coli* has suffered. For example, in *Acinetobacter baumannii*—which is one of the few species known to encode multiple *ppk1* genes in addition to *ppk2*—knockout of individual *ppk1*s had differing consequences on virulence [42]. *P. aeruginosa* possesses three PPK2s, only two of which, however, can synthesize polyP in vivo to compensate for loss of PPK1 [39,57]. These studies challenge the validity of generalizing certain aspects of *E. coli* polyP biology to organisms that possess PPK2s, since these enzymes may play species-specific niche roles. Furthermore, the presence of PPK2s in many of the current priority bacterial pathogens such as *P. aeruginosa* and *A. baumannii* highlights the importance of understanding PPK2 biology in the fight against bacterial infections (Table 1) [42,57].

## 3. PPK2 Enzymology

A large body of phylogenetic, structural, and biochemical data exists for PPK2 enzymes. Sequence analysis suggests that PPK2s evolved from a common P-loop kinase ancestor with thymidylate kinases [36], which catalyze the phosphorylation of thymidine 5′-onophosphate by ATP to yield the essential DNA precursor thymidine 5′-diphosphate. P-loop kinases are characterized by a conserved Walker-A motif (GxxxGK) that interacts with the β-phosphate of the bound nucleotide, and a Walker B motif that mediates binding to the nucleotide γ-phosphate via Mg^2+^ coordinated to a conserved carboxylate residue [60]. Phylogenetic analysis, combined with published biochemical data for select enzymes, revealed that PPK2s can be classified into three subfamilies based on nucleoside phosphate substrate preference: class I phosphorylate nucleoside diphosphates, class II phosphorylate nucleoside monophosphates, and class III can phosphorylate both nucleoside mono- or diphosphates [50] (Figure 1). Class III PPK2s, along with a common ancestor of class I and II PPK2, were likely the first to emerge, followed by diversification of class I and class II PPK2s. Since broad specificity is thought to be a property of primordial enzymes [61], the promiscuity of class III PPK2s supports the idea that this class is the closest to the original PPK2 ancestor [50].

### 3.1. PPK2 Crystal Structures and Catalytic Mechanism

**Figure 2:**
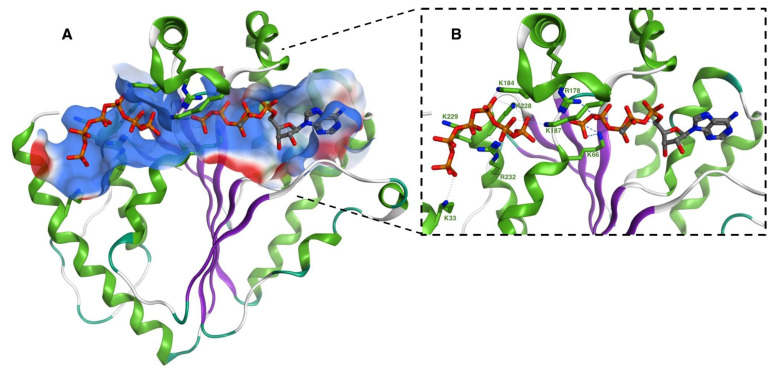
PPK2 substrate binding sites elucidated via co-crystallization with polyP and nucleotide ligand. *F. tularensis* PPK2 (green and purple) bound to polyP and the nucleotide AMP-PCH2PPP (PDB ID 5LLB) [53]. (**A**) Electrostatic surface representation of the polyP binding channel. Blue and red represent positive and negative charge, respectively. (**B**) Detailed view of the amino acid residues that mediate polyP binding. Interactions indicated by blue dashed lines. Figure created in Molecular Operating Environment.

To date, the crystal structures of PPK2s from seven different bacterial species have been published (Table 1). These include representatives from all three classes: class I: *Sinorhizobium meliloti* (PDB ID 3CZQ) and *Francisella tularensis* (PDB ID 4YEG); class II: *P. aeruginosa* PPK2C/PA3455 (PDB ID 3CZP); and class III: *Arthrobacter aurescens* (PDB ID 3RHF), *Meiothermus ruber* (PDB ID 5LC9), *Cytophaga hutchinsonii* (PDB ID 6ANG), and *Deinococcus radiodurans* (PDB ID 6AQE) [36,46,52,53]. These structures reveal a similar overall PPK2 architecture resembling that of thymidylate kinase [62]. Each PPK2 displays a three-layered α–β–α sandwich fold with a lid loop composed of two α-helices. An Mg^2+^ ion is frequently observed coordinated to the Walker A and/or Walker B motifs. The enzymes also share an internal substrate channel rich in positively charged residues that connect the polyP and nucleotide binding sites near the catalytic (Walker A) aspartate (Figure 2) [36,46,52,53]. Uniquely, the class II *P. aeruginosa* PPK2C has two fused PPK2 domains: a catalytically inactive N-terminal and an active C-terminal [36]. A comparison of the above crystal structures revealed a class-specific signature residue located after the Walker B motif: class I is asparagine (N153 in *S. meliloti* PPK2), class II is glycine (G367 in PA3455), and class III is glutamate (E137 in *C. hutchinsonii* PPK2) [46,63].

Several recent crystal structures of PPK2s complexed with polyP and/or nucleotides have provided invaluable insight into the substrate binding and reaction mechanisms of these enzymes. The class I *F. tularensis* PPK2 (*Ft*PPK2) has been crystallized in complex with polyP alone (PDB ID 5LL0) and both polyP and β,γ-methylene adenosine 5ʹ-pentaphosphate (AMP-PCPPP) (PDB ID 5LLB). The structures of class III *M. ruber* PPK2 (*Mr*PPK2) in complex with ADP and PPi (PDB ID 5MAQ), AMP (PDB ID 5LCD), ADP (PDB ID 5LDB), and ATP (PDB ID 5LD1) have also been solved. Likewise, class III *C. hutchinsonii* PPK2 (*Ch*PPK2) in complex with AMP (PDB ID 6ANG), ADP (PDB ID 6AN9), and guanosine 5-tetraphosphate (G4P) (PDB ID 6ANH) helps to further elucidate substrate specificity. *Ft*PPK2 co-crystallized with polyP revealed a polyP chain nine P_i_ units long bound between the protein core and the lid loop. The polyP is positioned by a cluster of lysine and arginine residues, which are conserved across all PPK2s [53] and reminiscent of the polyP tunnel observed in PPK1 [37] (Figure 2A). *Ft*PPK2 co-crystallized with both polyP and the nucleotide β,γ-methylene adenosine 5ʹ-triphosphate (AMP-PCP) yielded a structure in which two additional phosphoryl units were transferred onto AMP-PCP, yielding AMP-PCPPP. The nucleotide was bound on the right side of the Walker B motif, as predicted by a prior Nocek et al. study [36], with its five phosphate groups extending leftward towards the polyP channel, guided via coordination to the lid loop Mg^2+^ (Figure 2B). Although no electron density connecting the AMP-PCPPP to the polyP was visible, this spacing (∼7.0 Å) is consistent with the binding of six additional phosphoryl units. While intact polyP chains could not be observed in any of the *Mr*PPK2 structures, individual phosphate ions were observed in the region corresponding to the polyP channel in *Ft*PPK2. Alanine substitution of *Ft*PPK2 K66, R118, and R178 resulted in weaker polyP binding and dramatically reduced enzyme activity [53]. Similarly, alanine substitution the corresponding residues (K86, K258, and R262) in *Ch*PPK2 also markedly reduced activity. Nucleotide-bound structures of *Ch*PPK2 in complex with AMP, ADP, and G4P, as well as *D. radiodurans* PPK2 (*Dr*PPK2) in complex with ATP generally corroborated the binding orientations observed for *Ft*PPK2 [46]. Interestingly, two ATP molecules were observed in one active site for *Dr*PPK2 (PDB ID 6AQE). The first was bound in the typical nucleotide pocket, while the second was bound near the dimer interface [46]. Similarly, an Asn121Asp mutant of *Mr*PPK2 also contained two ATP, with the adenosine moiety of the second ATP contacting the adjacent protomer (PDB ID 5O6M) [53]. It is tempting to speculate that this type of secondary nucleotide binding may serve as a regulatory mechanism, perhaps by influencing oligomeric state, which could potentially help explain the substrate inhibition phenomenon observed for other PPK2s [57]. While PPK2s from various species have been observed to form dimers, trimers, tetramers, and octamers in solution [34,36,53,64], it is unclear how oligomerization affects activity or which oligomeric states are predominant in vivo.

Based on the described substrate-bound structures and complementary biochemical data, the PPK2 reaction mechanism is proposed to proceed via in-line nucleophilic attack by the nucleotide terminal phosphate oxygen on the terminal phosphorus atom of polyP [53]. The terminal phosphate groups of both substrates are coordinated by a bridging Lewis acidic Mg^2+^ (sometimes via a water molecule), which activates the polyP nucleophile. Several conserved positively charged residues located on the lid domain and the bottom of the active site (e.g., R208 and K81 of *Ch*PPK2) serve to stabilize the transferred phosphoryl group of polyP via hydrogen-bonding and charge compensation [46,53]. It is worth noting that this mechanism is distinct from that of PPK1, in which an autophosphorylated histidine residue mediates transfer of phosphate between the nucleotide and the polyP chain [4,37].

### 3.2. Class I PPK2 Enzymology

The following sections will summarize the biochemical characteristics of representative members of the class I, class II, and class III PPK2 families. The Kornberg lab conducted the first biochemical characterization of a class I PPK2 after having purified the enzyme to homogeneity from *P. aeruginosa* lysate. This enzyme (later called PPK2A/PA0141 to disambiguate from the other two *P. aeruginosa* PPK2s [39]) has a 75-fold preference for nucleoside diphosphate phosphorylation relative to polyP synthesis. Among nucleoside diphosphate acceptors, GDP is favored over ADP, as demonstrated by a roughly 2.5-fold lower Michaelis constant (K_m_). PPK2A shows no detectable AMP-phosphorylating activity; hence, it is a class I PPK2. Interestingly, Mg^2+^ is favored over Mn^2+^ for nucleoside diphosphate phosphorylation [34], but the inverse is true for polyP synthesis—Mn^2+^ elevates the PPK2A polyP synthesis rate to a level comparable to that of PPK1 [35]. A similar phenomenon was observed for the class I *Corynebacterium glutamicum* PPK2, which shows optimal activity with Mn^2+^, but remarkably exhibits a strong preference for polyP synthesis from ATP/GTP regardless of the cation provided [49]. A second class I *P. aeruginosa* enzyme PA2428 (dubbed PPK2B [39]) can readily phosphorylate ADP and GDP [36,65], and recent work from our lab has shown that this enzyme also exhibits exceptionally strong in vitro polyP synthesis activity from ATP, but only in the presence of an ATP regeneration system [57]. Other characterized class I enzymes include those of *S. meliloti*, *Agrobacterium tumefaciens*, Rhodopseudomonas palustris [36], *Mycobacterium tuberculosis* [54], and *F. tularensis* [52]. These enzymes fit the more traditional mold for class I PPK2s, demonstrating Mg^2+^-dependent ADP or GDP phosphorylation by polyP. An unusual member of the class I family is a PPK2 from *Silicibacter* (*Ruegeria) pomeroyi*—dubbed PPK3 by Nahalka and Patoprsty—that preferentially uses pyrimidine substrates [50,59]. Specifically, PPK3 catalyzed the polyP-dependent phosphorylation of CDP and UDP to CTP and UTP with roughly twofold higher activity compared to ADP/ATP and GDP/GTP [59]. Recently, two more class I enzymes were discovered to have the broadest nucleotide specificity to date. Both PPK2C from *Ralstonia eutropha* [66] and PPK2_AT_ from *Agrobacterium tumefaciens* were shown to phosphorylate ADP, GDP, CDP, dTDP, and UDP, in addition to performing the reverse reaction (polyP synthesis) from all five NTPs. This marks the first time PPK2s have been shown to use all the natural nucleotides—including thymidine—as substrates, highlighting the potential of these so-called universal PPK2s as tools for biotechnological NTP regeneration [66]. Specifically, universal PPK2s could be a boon for the regeneration of UTP and CTP required for the enzymatic synthesis of complex glycans [59,67,68], in addition to more traditional ATP regeneration systems (reviewed in [63]).

### 3.3. Class II PPK2 Enzymology

Class II PPK2s catalyze nucleoside monophosphate phosphorylation, and the first member of this family to be discovered was an enzyme from *Acinetobacter johnsonii* (*Aj*PPK2) initially termed polyP-AMP phosphotransferase (PAP) [69]. It was originally thought that this enzyme could only consume polyP, but subsequent experiments demonstrated that *Aj*PPK2 could also synthesize polyP from ADP. Indeed, *Aj*PPK2 had similar polyP synthesis kinetic parameters (K_m_ and V_max_) to *E. coli* PPK1. PolyP consumption favored high (100 mM) levels of MgCl_2_, whereas polyP synthesis was optimal at lower (20 mM) levels of MgCl_2_, once again suggesting that metal ion availability in the cell may serve to regulate PPK2 reaction preference [43]. *Aj*PPK2 was later shown to phosphorylate GMP and dAMP, but not CMP, UMP, or IMP [44]. Uniquely, the majority of PPK2s in the class II family contain two fused PPK2 domains and are thus roughly double the size of class I or III enzymes. However, the class II category contains both one-domain and two-domain PPK2s; therefore, prediction of biochemical activity based solely on the number of domains does not always hold true [50]. Both *Aj*PPK2 and *P. aeruginosa* PPK2C (PA3455) are two-domain PPK2s. PPK2C exhibited optimal activity when phosphorylating AMP to ADP, but it could also phosphorylate GMP, dAMP, dGMP, IMP, and XMP. Mg^2+^ was the most effective cofactor, while Co^2+^ and Ni^2+^ yielded weak activity, and Mn^2+^ and Ca^2+^ had no activity. Unlike *Aj*PPK2, PPK2C had no detectable polyP synthesis activity under the conditions tested [36]. Characterized one-domain class II PPK2s include that of *Myxococcus xanthus* [56,70] and *Bacillus cereus* [47]. In contrast to *Aj*PPK2 and *P. aeruginosa* PPK2C, *M. xanthus* PPK2/PAP had twofold higher activity in the presence of Mn^2+^ compared to Mg^2+^ [56].

### 3.4. Class III PPK2 Enzymology

Class III is the most recently discovered PPK2 subfamily, capable of phosphorylating either nucleoside mono- or diphosphates. Based on their phylogenetic prediction of this new class of enzymes, Motomura and colleagues first validated PPK2s from *M. ruber*, *Meiothermus silvanus*, Deinococcus geothermalis, Thermosynechococcus elongatus, and *D. radiodurans* as capable of polyP-driven ATP synthesis from AMP [50]. Nocek et al. added two more class II enzymes to the list: *A. aurescens* (*Aa*PPK2) and *C. hutchinsonii* (*Ch*PPK2). *Ch*PPK2, *Dr*PPK2, and *Aa*PPK2 all showed significant activity with Mg^2+^, Mn^2+^, Ca^2+^, Co^2+^, and Ni^2+^ [46], indicating that class III PPK2s appear to accept a broader range of metal ion cofactors relative to class I and II enzymes. As expected, the polyP synthesis activity of *Aa*PPK2, *Ch*PPK2, and *Dr*PPK2 was at least three orders of magnitude lower than polyP-dependent phosphorylation of NMPs or NDPs. While *M. ruber* PPK2 was capable of using both purine and pyrimidine bases [50], *Aa*PPK2, *Ch*PPK2, and *Dr*PPK2 showed no activity with pyrimidines. However, the latter three enzymes showed significant activity with dAMP and dADP, suggesting that these PPK2s may contribute to the synthesis of DNA precursors. Interestingly, these three class III PPK2s in addition to the class I SMc0218 and class II PA3455 showed weak adenylate kinase activity (conversion of ADP to AMP and ATP) [46]. An outstanding question surrounding class III PPK2s is whether the conversion of nucleoside monophosphates to nucleoside triphosphates involves a direct (pyrophosphorylation) or sequential transfer of phosphate groups. A recent study by Ogawa et al. concluded that a significant amount (30–80%, depending on the PPK2 tested) of ATP produced from AMP occurs via pyrophosphorylation, and the rest occurs via step-by-step monophosphorylation by separate P_i_ molecules [51]. While the authors of this study concede that their methodology could not exclude the possibility of some sequential monophosphorylation being mistaken for pyrophosphorylation, previous reports that PPK2 polyP utilization is processive [34,35,69]—i.e., the polyP chain is not released by the enzyme between reactions—support the possibility that multiple P_i_ units can be transferred from polyP at once. Regardless of the reaction mechanism, class III enzymes offer a streamlined means of recycling both AMP and ADP and are thus beginning to supplant class I enzymes for use in biotechnological ATP regeneration systems [63].

### 3.5. Other PPK2 Activities

Some PPK2 reaction products do not fit squarely within the categories described above. Moreover, the catalytic activities defining the three PPK2 classes should be considered preferences, rather than absolute definitions, as several class I and class II enzymes were recently shown to be capable of AMP phosphorylation to ATP in the presence of large (500 µg/mL) amounts of enzyme [63,71]. Several studies have also described unusual nucleoside phosphate species produced by PPK2s. Representative PPK2s from all three classes (*Sm*PPK2, *Ft*PPK2, *Aj*PPK2, and *Mr*PPK2) were shown to synthesize detectable levels of adenosine tetraphosphate (AP4) and adenosine pentaphosphate [71]. Similarly, the class III PPK2 from *Delftia tsuruhatensis* also makes AP4 [51]. Most recently, the class I *A. tumefaciens* PPK2 has been shown to catalyze the formation of oligophosphorylated nucleosides from GDP, CDP, dTDP, and UDP, with nona-phosphorylated adenosine being the largest product observed [45]. Crystallization of *Ft*PPK2 with AMP-PCP and polyP resulted in a structure that clearly showed two extra phosphate groups added to AMP-PCP to yield AMP-PCPPP (Figure 2), though this species could not be detected in free solution enzyme reactions [53]. It remains to be determined whether these unorthodox PPK2 products are merely artifacts of in vitro reaction conditions, or if they also serve physiological functions in microbial systems. Ultraphosphates (branched polyphosphates) were recently shown to be a useable substrate for the enzyme alkaline phosphatase [72], raising the intriguing possibility that PPK enzymes could also consume, synthesize, or modify branched polyP chains. Moreover, cyclic polyphosphates (metaphosphates) have been detected in the polyP granules of the bacterium *Xanthobacter autotrophicus* [73]. Whether PPK enzymes play a role in the synthesis or utilization of these cyclic polyP molecules in bacteria remains an area ripe for future exploration.

## 4. Roles of PPK2s in Bacterial Physiology and Virulence

**Figure 3:**
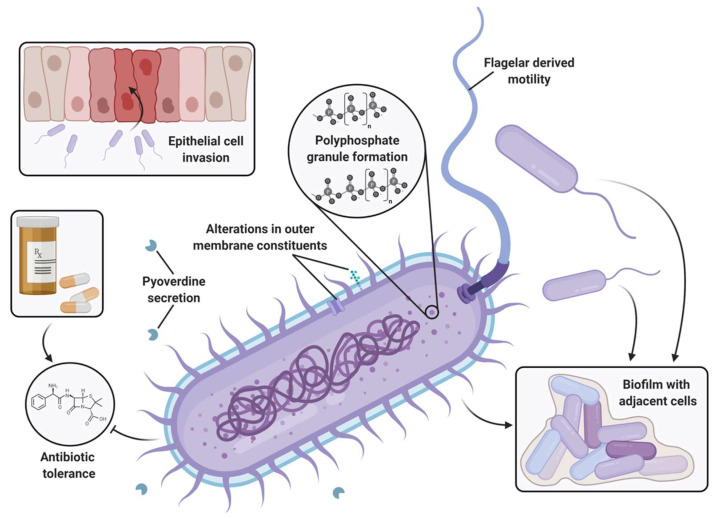
Schematic of bacterial virulence phenotypes linked to PPK2 enzymes. Figure created using BioRender.

Although generally understudied compared to their PPK1 counterparts, PPK2 enzymes are being increasingly implicated as important regulators of bacterial physiology and virulence. The following sections will summarize the contributions of PPK2 enzymes to bacterial homeostasis, stress response, biofilms, cell invasion, and antibiotic susceptibility, as illustrated in Figure 3.

### 4.1. Bacterial Homeostasis and Stress Response

*P. aeruginosa* has among the best-characterized PPK2 physiology to date. The Kornberg lab’s initial studies of *P. aeruginosa* PPK2A (PA0141) revealed that expression of this enzyme was induced over 100-fold at stationary phase [34], suggesting its importance for stress survival. It is important to note that *P. aeruginosa* encodes three PPK2 enzymes: PPK2A (PA0141), PPK2B (PA2428), and PPK2C (PA33455), in addition to PPK1 (PA5242). Work by Racki et al. demonstrated that a quadruple knockout strain of *P. aeruginosa* PA14 lacking all four PPK enzymes (∆*polyP*) was defective in cell cycle exit, which is dependent on the formation of organized polyP granules [39]. Importantly, there was no significant difference in growth kinetics between WT and ∆*polyP* PA14 [57]. As expected, the ∆*polyP* strain did not produce any detectable polyP, but a strain that retained functional PPK2A (∆*ppk1*∆*ppk2B*∆*ppk2C*) still formed polyP granules and exhibited near-normal cell cycle exit. In contrast, individual presence of chromosomally encoded *ppk2B* or *ppk2C* was insufficient for polyP granule formation. Interestingly, when *ppk2B* was re-introduced on a plasmid, it was sufficient to generate polyP granules [39], suggesting that PPK2B could also compensate for the loss of PPK1, provided that conditions are suitable for expression or otherwise full induction of enzymatic activity.

In *Campylobacter jejuni*, *ppk2* deletion resulted in decreased polyP-dependent GTP synthesis. This yielded an increased intracellular ATP:GTP ratio but had no effect on polyP or guanosine tetraphosphate (ppGpp) alarmone levels. *ppk2* deletion was also associated with significant variations in transcriptional abundance. For example, expression of the gene encoding SpoT—an enzyme which synthesizes and hydrolyzes ppGpp—was upregulated over 25-fold in the ∆*ppk2* mutant, which the authors hypothesize may serve as a compensatory mechanism to keep ppGpp levels low. The *C. jejuni ppk2* mutant was also defective in survival during stationary phase nutrient starvation, osmotic stress, and aerobic stress [48]. A *ppk2* mutant strain of *Mycobacterium smegmatis* displayed a similarly elevated ATP:GTP ratio, indicating that PPK2 modulates nucleotide pools in vivo. *M. smegmatis* PPK2 has been shown to directly bind to nucleoside diphosphate kinase (Ndk) to direct its activity towards synthesis of GTP as opposed to CTP or UTP, which may contribute to the elevated ATP:GTP ratio. The *M. smegmatis ppk2* mutant was also compromised in survival following thermal, acidic, and hypoxic stress. Double knockout of both *ppk2* and *ppk1* in *M. smegmatis* appears to be lethal [54], and repeated attempts to construct a *C. jejuni ppk1*–*ppk2* double knockout were also unsuccessful [48], suggesting a critical role of polyP in these species. In *Mycobacterium tuberculosis*, a *ppk2* transposon mutant accumulated significantly more intracellular polyP despite also exhibiting significantly reduced *ppk1* gene expression [41]. The *M. tuberculosis ppk2* mutant also accumulated less glycerol-3-phosphate, an important phospholipid precursor, and 1-deoxy-xylulose-5-phosphate, a peptidoglycan biosynthetic metabolite [74].

Remarkably, *R. eutropha*—an environmental bacterium known for its ability to grow using hydrogen as the electron donor—encodes seven *ppk* genes: two *ppk1* and five *ppk2* genes. At least three of these enzymes (PPK1a, PPK2b, and PPK2c) contribute to polyP synthesis and at least four of them (PPK1a, PPK2c, PPK2d, and PPK2e) colocalize with polyP granules in vivo [40]. Overexpression of plasmid-encoded *ppk2c* in an *R. eutropha* polyP-free background (∆*ppk-all*) lacking all seven PPKs was sufficient to generate large polyP granules [75]. To study the contribution of these enzymes to *R. eutropha* physiology, Rosigkeit et al. tested the effects of various stressors on the ∆*ppk-all* strain. Surprisingly, the ∆*ppk-all* mutant showed no defects in heat shock survival, oxidative stress survival, or motility—all phenotypes that have previously been linked to PPK1 in *E. coli* [19,33,58]. The ∆*ppk-all* mutant did display a marginal growth defect in phosphate-depleted media, indicating a role of polyP and PPK1/2s in *R. eutropha* phosphate storage [58]. Given the remarkable number of PPK2 enzymes in *R. eutropha*, it seems likely that additional roles for these enzymes remain to be discovered. The marked discrepancy in behavior between *R. eutropha* and *E. coli ppk* mutants in the face of stressors also serves to caution against broad generalizations of polyP functions between species, particularly when multiple PPK1 or PPK2 isoforms are present.

### 4.2. Biofilms

The Kornberg lab’s initial discovery that *P. aeruginosa* PPK2A preferentially synthesizes GTP—a precursor for making the alginate found in Pseudomonad biofilms—led to speculation that PPK2A was important for biofilm formation [34]. Our group provided the first experimental evidence in support of this hypothesis, demonstrating that a ∆*ppk2A*∆*ppk2B*∆*ppk2C* mutant of *P. aeruginosa* PA14 was attenuated in biofilm formation relative to the WT, roughly mirroring the biofilm defect observed in the ∆*ppk1* mutant. Biofilm formation of the ∆*polyP* strain lacking all four *ppk* genes was even further attenuated compared to ∆*ppk2A*∆*ppk2B*∆*ppk2C* or *∆ppk1* [57]. Further research is needed to decipher the individual contribution of PPK2A, PPK2B, and PPK2C in biofilm formation. Similarly, a *ppk2* transposon mutant of *M. tuberculosis* strain CDC1551 was shown to be defective in pellicle biofilm formation [74]. However, in a different study, *ppk2* deletion in *M. tuberculosis* strain H37Rv did not appear to affect biofilm formation upon visual inspection, although the authors did not quantify the biofilm [55]. This disagreement could be explained by the use of different parental strains, or the differing means of *ppk2* gene inactivation. Surprisingly, a *ppk2* mutant of *C. jejuni* formed significantly more biofilm than the WT [48]. Caution must therefore be taken when generalizing the relationship between PPK2 and biofilm from one species or strain to another.

### 4.3. Virulence Factors and Invasion

Virulence factors are broadly defined as bacterial products or phenotypes that enable colonization, replication, or dissemination within the host organism [76]. Motility—whereby flagella or pili drive bacterial locomotion—is a necessity for the virulence of many species, and *ppk2* deletion (∆*ppk2A*∆*ppk2B*∆*ppk2C*) has been shown to reduce swimming motility in *P. aeruginosa* [57]. Likewise, secreted toxins such as the siderophore pyoverdine are another class of virulence factor that frequently underpin *P. aeruginosa* infections [77]. We showed that relative to the *P. aeruginosa* ∆*ppk1* strain, the ∆*polyP* strain was further attenuated in pyoverdine production, thus implicating PPK2 enzymes in pyoverdine virulence. The ∆*polyP* strain also demonstrated a further reduction of *P. aeruginosa* virulence towards the nematode *Caenorhabditis elegans* relative to the ∆*ppk1* strain, highlighting the importance of *P. aeruginosa* PPK2s in an animal model of infection for the first time [57]. Further studies to dissect the individual contributions of each *ppk2* isoform to these phenotypes may help elucidate the mechanistic links between polyP metabolism and virulence.

While *P. aeruginosa* is primarily an extracellular pathogen, PPK2 enzymes have also been extensively linked to the invasion and survival of the intracellular pathogens *C. jejuni*, *M. tuberculosis*, and *F. tularensis*. A *C. jejuni ppk2* mutant was defective in invasion and intracellular survival in cultured human intestinal epithelial cells. Furthermore, *C. jejuni* ∆*ppk2* was also significantly impaired in the colonization of day-old chickens [48]. These defects may be due to changes in outer membrane constituents (proteins, lipids, and polysaccharides), alterations of which in *C. jejuni* ∆*ppk2* have been shown to attenuate its invasion [78]. In *M. tuberculosis*, *ppk2* mutation or downregulation impaired growth and survival in macrophages [41,54,55]. Moreover, macrophages infected with ∆*ppk2* bacteria secreted increased levels of proinflammatory cytokines [41], which culminated towards defects in colonization following bacterial challenge in both murine and guinea pig models of infection [41,55]. The lungs of guinea pigs infected with ∆*ppk2 M. tuberculosis* had fewer and smaller tubercles relative to the WT and *ppk2*-complemented controls [55]. Similarly, deletion of *ppk2* (FTT_1564) in *F. tularensis* abolished polyP accumulation, and resulted in defective intracellular growth in macrophages and attenuated infection of mice [79].

### 4.4. Antibiotic Sensitivity

With the world facing a rising tide of antibiotic-resistant bacterial infections, an intriguing development has been the connection between polyP, PPKs, and antibiotic sensitivity. A ∆*ppk2A* strain of *P. aeruginosa* PAO1 showed increased susceptibility to several clinically relevant antibiotics, including imipenem and ciprofloxacin [80]. Likewise, an *F. tularensis ppk2* mutant was more susceptible to three distinct classes of antibiotics: the aminoglycosides streptomycin and gentamicin, tetracyclines tetracycline and doxycycline, and the fluoroquinolone ciprofloxacin [52]. *C. jejuni* ∆*ppk2* exhibited increased susceptibility to erythromycin and ciprofloxacin, which are considered drugs of choice for treating *C. jejuni* infections [48]. *M. tuberculosis ppk2* mutation yielded increased susceptibility to the L,D-transpeptidase inhibitor antibiotic meropenem, which could potentially be explained by the reduced transcription of L-D-transpeptidase genes observed in the *ppk2* mutant [74]. In contrast, two independent reports showed that *M. tuberculosis ppk2* mutants exhibit a fourfold increase in the minimum inhibitory concentration of isoniazid [41,55]. While the first four studies suggest the exciting possibility of inhibiting PPK2 enzymes to extend the lifespan of existing antibiotics, the isoniazid example behooves careful testing of potential PPK2 inhibitors in combination with antibiotics prior to clinical use.

## 5. Therapeutic Potential: PPK2 Inhibitors

Given the important roles of PPK2s in bacterial virulence and infection, these enzymes have been suggested as attractive drug targets. However, relatively few PPK2 inhibitors have been documented in vitro, and to the best of our knowledge, only one has been validated for PPK2 inhibition activity in vivo. A summary of published PPK2 inhibitors is provided in Table 2. Using a luciferase-based activity assay, Singh et al. screened a 2300-member National Cancer Institute small molecule library against purified maltose binding protein (MBP)-tagged *M. tuberculosis* PPK2. This screen identified the heteroaromatic compounds NSC 35676, NSC 30205, NSC 345647, and NSC 9037 as inhibitors of PPK2 at low-micromolar concentrations [55]. More recently, a series of (bis)phosphonic acid-derived PPK2 inhibitors have been developed [81]. Initially, three aryl phosphonate inhibitor molecules were co-crystallized with *C. hutchinsonii* PPK2, revealing the value of a (bis)phosphonate-based scaffold for PPK2 inhibition since this moiety was observed to competitively occupy the polyP channel [46]. A follow-up study synthesized 32 (bis)phosphonic acid derivatives, the best of which inhibited *C. hutchinsonii* PPK2 with an IC_50_ of approximately 60 µM [81] (Table 2). The most potent PPK2 inhibitor documented to date is a G-quadruplex DNA aptamer that specifically binds *M. tuberculosis* PPK2 (IC_50_ = 40 nM) [64]. However, none of the aforementioned studies tested their inhibitors in bacteria. We recently discovered that the polyphenolic molecule gallein inhibits *P. aeruginosa* PPK1 and PPK2s in vitro at low-micromolar doses. Importantly, we showed that gallein also inhibits both PPK1 and PPK2s in the bacterial cell, phenocopying the ∆*polyP* strain (knockout lacking all four *ppk* genes) to attenuate polyP accumulation, biofilm formation, pyoverdine production, and virulence in *C. elegans*. Gallein also synergized with the antibiotics tetracycline and ciprofloxacin to improve *C. elegans* survival following *P. aeruginosa* infection [57,82]. In a follow-up study, we also demonstrated that gallein inhibits *A. baumannii* and *K. pneumoniae* PPK2s in vitro [42] (Table 2).

## 6. Conclusions and Future Outlook

Our understanding of PPK2s has expanded rapidly since the discovery of this new class of enzymes nearly twenty years ago. The multitude of biochemical studies on purified PPK2s from diverse bacteria has provided a characterization of PPK2 enzymology that is arguably more thorough than that of PPK1s. However, where PPK2 research still lags behind PPK1 research is in studies of bacterial physiology. While *ppk2* deletion has been shown to influence stress response and virulence in several species, the resultant phenotypes appear to be less predictable than those observed in *ppk1* knockouts. Moreover, in species that possess both *ppk1* and *ppk2* genes—which include the priority pathogens *A. baumannii*, *K. pneumoniae*, and *P. aeruginosa*—the interplay between PPK1, PPK2, polyP, and downstream virulence phenotypes remains an area in need of further clarification. While the number of validated PPK2 inhibitors also trails in comparison to the number of validated PPK1 inhibitors, the abundance of structural and biochemical data now available for PPK2s suggests that the time is ripe for inhibitor development. Recent co-crystal structures of PPK2s in complex with inhibitors provide a promising roadmap for future structure-based drug design. Likewise, the availability of substrate-bound PPK2 structures has enhanced the prospects of in silico chemical library screening for inhibitors targeting either the nucleotide site or the polyP channel. As the role of PPK2s and polyP in bacterial physiology and virulence becomes more and more apparent, so too does the value of PPK2 enzymes as drug targets. PPK2 inhibitors, therefore, seem poised to become an important weapon in the fight against antibiotic-resistant bacteria.

## Figures and Tables

**Table 1:** Summary of bacterial species with characterized PPK2 enzymes.

Species	Number of PPK2s	PPK2 Class	PDB ID	Reference
*Acinetobacter baumannii*	1	II		[42]
*Acinetobacter johnsonii*	1	II		[43,44]
*Agrobacterium tumefaciens*	1	I		[36,45]
*Arthrobacter aurescens*	1	III	3RHF	[46]
*Bacillus cereus*	1	II		[47]
*Campylobacter jejuni*	1	I		[48]
*Corynebacterium glutamicum*	2	I		[49]
*Cytophaga hutchinsonii*	1	III	6ANG, 6ANH, 6ANQ, 6AUO, 6AN9, 6B18	[46]
*Deinococcus geothermalis*	1	III		[50]
*Deinococcus radiodurans*	1	III	6AQE, 7NMJ, 7BMM	[46,50]
*Delftia tsuruhatensis*	1	III		[51]
*Francisella tularensis*	1	I	4YEG, 5LLB, 5LL0, 5LLF	[52,53]
*Klebsiella pneumoniae*	1	I		[42]
*Meiothermus ruber*	1	III	5LC9	[50,53]
*Meiothermus silvanus*	1	III		[50]
*Mycobacterium smegmatis*	1	I		[54]
*Mycobacterium tuberculosis*	1	I		[41,55]
*Myxococcus xanthus*	1	II		[56]
*Pseudomonas aeruginosa*	3	I (PPK2A/PA0141)		[34,57]
		I (PPK2B/PA2428)		[39,57]
		II (PPK2C/PA3455)	3CZP	[36]
*Ralstonia eutropha*	5	Not yet classified		[40,58]
*Rhodopseudomonas palustris*	1	I		[36]
*Ruegeria pomeroyi*	1	I		[59]
*Sinorhizobium meliloti*	3	I	3CZQ, 6DZG	[36]
*Thermosynechococcus elongatus*	1	III		[50]

**Table 2:** Chemical structures and activities of PPK2 inhibitors.

Inhibitor	Structure	Reaction Tested	Inhibition Potency	Reference
NSC 35676	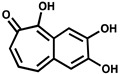	ATP synthesis from ADP	>80% inhibition at 100 µM of *M. tuberculosis* PPK2–MBP fusion	[55]
NSC 30205	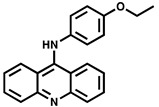	ATP synthesis from ADP	>80% inhibition at 100 µM of *M. tuberculosis* PPK2–MBP fusion	
NSC 345647	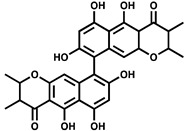	ATP synthesis from ADP	>80% inhibition at 100 µM of *M. tuberculosis* PPK2–MBP fusion	
NSC 9037	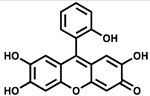	ATP synthesis from ADP	>80% inhibition at 100 µM of *M. tuberculosis* PPK2–MBP fusion	
11f	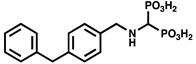	ATP synthesis from ADP	IC_50_ = 60.2 µM for *C. hutchinsonii* PPK2	[81]
11g	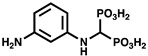	ATP synthesis from ADP	IC_50_ = 70.5 µM for *C. hutchinsonii* PPK2	
11i	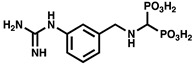	ATP synthesis from ADP	IC_50_ = 58.0 µM for *C. hutchinsonii* PPK2	
14b	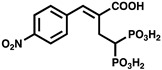	ATP synthesis from ADP	IC_50_ = 85.4 µM for *C. hutchinsonii* PPK2	
Gallein	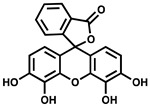	ATP synthesis from ADP	IC_50_ = 16 µM for *P. aeruginosa* PPK2A	[57]
		PolyP synthesis from ATP	IC_50_ = 20 µM for *P. aeruginosa* PPK2B	
		ADP synthesis from AMP	IC_50_ = 165 µM for *P. aeruginosa* PPK2C	
		ADP synthesis from AMP	IC_50_ = 40.7 µM for *A. baumannii* PPK2	[42]
		ATP synthesis from ADP	IC_50_ = 50.4 µM for *K. pneumoniae* MBP-PPK2	
Aptamer G9	N/A	ATP synthesis from ADP	IC_50_ = 39.3 nM for *M. tuberculosis* PPK2	[64]

## Data Availability

Not applicable.

## Abbreviations

*Aj*PPK2	*Acinetobacter johnsonii* PPK2
AMP-PCP	β,γ-methylene adenosine 5ʹ-triphosphate
AMP-PCPPP	β,γ-methylene adenosine 5ʹ-pentaphosphate
AP4	adenosine tetraphosphate
*Ch*PPK2	*Cytophaga hutchinsonii* PPK2
*Dr*PPK2	*Deinococcus radiodurans* PPK2
*Ft*PPK2	*Francisella tularensis* PPK2
G4P	guanosine 5′-tetraphosphate
IC_50_	half-maximal inhibition constant
K_m_	Michaelis constant
MBP	maltose binding protein
*Mr*PPK2	*Meiothermus ruber* PPK2
Ndk	nucleoside diphosphate kinase
PAP	polyP-AMP phosphotransferase
P_i_	inorganic phosphate
polyP	polyphosphate
ppGpp	guanosine tetraphosphate
PPK	polyphosphate kinase
*Sm*PPK2	*Sinorhizobium meliloti* PPK2
V_max_	maximum enzyme velocity
